# Growth and characterization of thin Cu-phthalocyanine films on MgO(001) layer for organic light-emitting diodes

**DOI:** 10.1186/1556-276X-7-650

**Published:** 2012-11-26

**Authors:** Yu Jeong Bae, Nyun Jong Lee, Tae Hee Kim, Hyunduck Cho, Changhee Lee, Luke Fleet, Atsufumi Hirohata

**Affiliations:** 1 Department of Physics, Ewha Womans University, Seoul, 120-750, South Korea; 2 School of Electrical Engineering and Computer Science, Seoul National University, Seoul, 151-744, South Korea; 3 Department of Electronics, The University of York, York, YO10 5DD, UK

**Keywords:** Organic light-emitting diode, Fe/MgO(001) epilayers, Cu-phthalocyanine, Thermal stability, Hybrid organic spintronics hybrid structures, 68.55-a, 81.07Pr, 81.05.Fb

## Abstract

Surface morphology and thermal stability of Cu-phthalocyanine (CuPc) films grown on an epitaxially grown MgO(001) layer were investigated by using atomic force microscope and X-ray diffractometer. The (002) textured β phase of CuPc films were prepared at room temperature beyond the epitaxial MgO/Fe/MgO(001) buffer layer by the vacuum deposition technique. The CuPc structure remained stable even after post-annealing at 350°C for 1 h under vacuum, which is an important advantage of device fabrication. In order to improve the device performance, we investigated also current-voltage-luminescence characteristics for the new top-emitting organic light-emitting diodes with different thicknesses of CuPc layer.

## Background

For charge injection into organic semiconductor (OSC) devices, oxide materials have been widely utilized. In general, indium thin oxide (ITO) is used as a transparent conducting electrode for optoelectronic devices which enable light to be emitted from the bottom of the structure. The work functions and electronic properties of ITO surfaces and, consequently, the interface electronic properties of contacts to OSC have become important issues to improve the organic light-emitting diode (OLED) device performance [1]. Different chemical and physical treatments have been investigated to control the work function of an anode ITO electrode. In order to control efficiently the charge injection and transport in OSC devices, a novel approach by introducing a thin oxide layer between an anode and a hole-transport layer (HTL) in organic light-emitting diodes (OLEDs) has gotten considerable attentions recently [2-4]. After the first report of the role of ITO as a charge generation layer (CGL) [5], several other studies followed to demonstrate the effect of CGL based on oxide films, such as MoO_3_[6], V_2_O_5_[7], and WO_3_[1]. The improved device efficiency was attributed to the generation of holes which could reduce the charge injection barriers at organic semiconductor interfaces upon application of an electric field.

Considering the monolithic integration of an OLED on silicon or organic thin film transistors as well as spin valves, a thin epitaxial MgO(001) layer as the CGL in OLEDs might be interesting to investigate for magneto-optoelectronic applications. The crystalline MgO(001) layer grown on Fe(001) is well known as a tunnel barrier for the fully epitaxial Fe(001)/MgO(001)/Fe(001) tunnel junctions with a huge magnetoresistance (MR) value at room temperature (RT) due to a band symmetry filtering effect [8,9]. Remarkable improvements have been recently achieved in the field of organic spin valve devices [10,11]. However, according to our knowledge, up to now the investigation on the MgO-based organic spin valves with a huge MR is not reported yet.

In order to understand the effect of an epitaxial MgO(001) layer on the charge generation mechanism of OLED devices, a preliminary investigation to fabricate the hybrid multilayer films with sharp amorphous or crystalline interfaces is required. However, the growth of organic thin films beyond the metal or oxide contact was challenging; and a number of problems had to be solved, especially to obtain smooth and ordered molecular monolayer of the organic materials suitable for applications of organic spintronic devices [12]. Therefore, in this work, we investigated the growth of organic-inorganic hybrid multilayer structures for Si(001) substrate-based OLED devices. In particular, we focused on the growth of layer-structured HTL Cu-phthalocyanine (CuPc) and characterization of its surface morphology after heat-treatment under vacuum, since CuPc is one of the most popular OSC with high thermal and chemical stability suitable for thin film organic devices [13]. Thus, it has been also widely used in optoelectronic devices [14].

Additionally, a new top-emitting OLED (TOLED) structure, which is formed on an opaque Si(001) substrate and an epitaxial MgO(001)/Fe(001)/MgO(001) bottom electrode so that light can emit from the thin Al top electrode, was investigated. Our TOLED design includes a semi-transparent cathode Al, a stack of conventional organic electroluminescent layers, and a thin CuPc film to enhance the hole injection into the electroluminescent layers.

We expect that our new approach could open up the door toward the development of multifunctional architectures for future device technology.

## Methods

We used a ultra-high vacuum (UHV)-molecular beam epitaxy film evaporation system to stack successively inorganic elements beyond chemically etched p-type Si(001) wafers. The base pressure of the UHV-MBE chamber was lower than approximately 2 × 10^−10^ Torr. The epitaxial MgO(001)/Fe(001)/MgO(001) multilayers were formed at 250°C with low deposition rate of 0.003 nm/s. During the deposition the pressure was kept lower than 3 × 10^−9^ Torr.

In order to investigate the thin CuPc film growth mode and its structural properties, 20-nm-thick CuPc plane films were deposited at RT beyond Si(001)/8.0 nm MgO/15 nm Fe/1.8 nm MgO by high vacuum thermal evaporation (base pressure at approximately 2 × 10^−7^ Torr). It should be noted that air-exposure on the MgO top surface is inevitable during the sample transfer from the UHV-MBE chamber to the HV-thermal evaporator.

**Figure 1:**
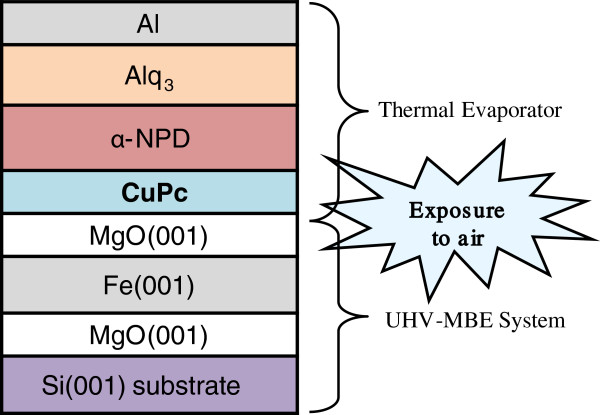
**Schematic structure of our OLED devices.** MgO/Fe/MgO(001) structures were prepared beyond a chemically etched Si(001) wafer by using UHV-MBE technique, then followed by deposition of CuPc/α-NPD/Alq_3_/Al multilayers at room temperature by using thermal evaporation in high vacuum.

Figure 1 presents the schematic structure of multi layer TOLED. The 20-nm-thick Al cathode was deposited on the OLED layer structure without incurring damage to the underlying active organic emissive layer: CuPc as a hole-injection layer, *N*,*N*^′^-di(1-naphthyl)-*N*,*N*^′^-diphenylbenzidine (α-NPD) as a hole-transport layer and 8-*tris*-hydroxyquinoline aluminum (Alq_3_) as a light-emitting layer. During the deposition of the organic films, the pressure was kept no more than 2 × 10^−6^ Torr, and the deposition rate was fixed at 0.03 nm/s. During deposition, the layer thickness was obtained by a quartz thickness monitor and verified after deposition by a step-profiler. The names of the TOLEDs with their structural information are listed in Table 1.

**Table 1:** Sample description

	
	** Substrate and layers**
S1	Si(001)\SiO_2_\150 nm Al\10 nm CuPc\60 nm α-NPD\70 nm Alq_3_\20 nm Al
S2	Si(001)\8 nm MgO\15 nm Fe\1.8 nm MgO\10 nm CuPc\60 nm α-NPD\70 nm Alq_3_\20 nm Al
S3	Si(001)\8 nm MgO\15 nm Fe\10 nm CuPc\60 nm α-NPD\70 nm Alq_3_\20 nm Al
S4	Si(001)\SiO_2_\15 nm poly-Fe\10 nm CuPc\60 nm α-NPD\70 nm Alq_3_\20 nm Al
S5	Si(001)\8 nm MgO\15 nm Fe\1.8 nm MgO\15 nm CuPc\60 nm α-NPD\70 nm Alq_3_\20 nm Al
S6	Si(001)\8 nm MgO\15 nm Fe\1.8 nm MgO\5 nm CuPc\60 nm α-NPD\70 nm Alq_3_\20 nm Al
S7	Si(001)\8 nm MgO\15 nm Fe\1.8 nm MgO\1 nm CuPc23\60 nm α-NPD\70 nm Alq_3_\20 nm Al

For the Si(001)/MgO/Fe/MgO/CuPc hybrid multilayers before and after vacuum annealing for 1 h in the temperature range from 100°C to 250°C, the structural characteristics were examined by using X-ray powder diffraction technique (XRD) and atomic force microscope (AFM). In order to analyze the microstructure of these hybrid multilayer interfaces, cross-sectional samples for transmission electron microscope (TEM) were prepared using conventional mechanical polishing and dimpling techniques. All the images were obtained using a double aberration corrected JEOL FS2200 TEM (JEOL Ltd., Tokyo, Japan) with atomic resolution. A new specimen preparation process with minimum damage onto the organic layer was developed. We used mechanical thinning, followed by precision ion-beam polisher system or (PIPS) with very short time to clean the surface. We have also minimized the damage during the TEM observation by reducing the e-beam exposure time. In addition we have confirmed the presence of the CuPc by energy dispersive X-ray scan using the scanning TEM observation mode.

The current-voltage-luminance (*I*-*V*-*L*) characteristics were investigated by using a Keithley 236 source-measure unit and a Keithley 2000 multimeter equipped with a photomultiplier tube through an ARC 275 monochromator (Keithley Instruments Inc., Cleveland, OH, USA). The external quantum efficiency of the electroluminescence (EL), defined as the ratio of the emitted photons to the injected electric charges, was calculated from the EL intensity measured by using a calibrated Si photodiode placed at a normal angle to the device’s surface.

## Results and discussion

**Figure 2:**
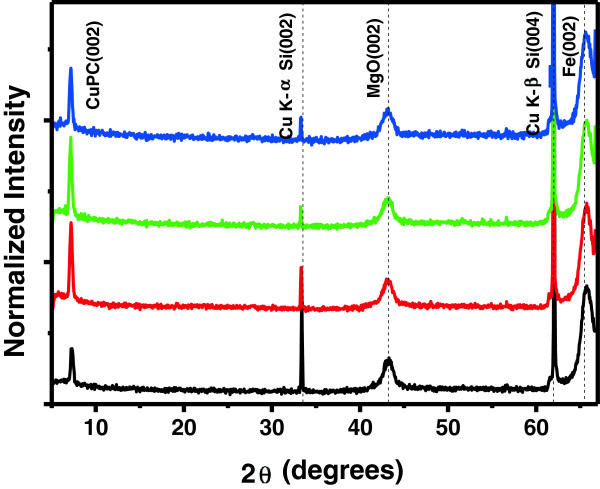
**X-ray diffraction *θ*-2*θ *scans with Cu Kα radiation for the 20-nm-thick CuPc films before and after thermal treatment.**

The main goal of this study was to obtain an enhancement of the thermal durability of the OLEDs. Particular attention was given to the engineering of charge injecting contacts by introducing the epitaxial thin MgO(001) layer. Figure 2 shows an important enhancement of thermal stability of the 20-nm-thick CuPc film grown the MgO(001) layer. Black color represents the result obtained before annealing, while red, green and blue colors correspond to the results for the sample after 1 h vacuum annealing at 250°C, 300°C and 350°C, respectively. The dashed lines represent CuPc, MgO and Fe peak positions from the powder diffraction file. After 1 h of vacuum annealing in the temperature range from 150°C to 350°C, a strong diffraction peak, corresponding to the (002) lattice plane of β-phase CuPc, persists at 2*θ* = 7.15° which is quite close to the position expected from the reference data. The peak intensity increases as the annealing temperature increases up to 250°C, while the intensity decreases after the annealing at 350°C. These XRD patterns indicate that the Fe and MgO buffer layers are strongly (001) textured, only the (002) diffraction peaks of bcc Fe and fcc MgO are observed. Consequently, the CuPc films deposited on the epitaxial Fe/MgO(001) buffer layer are also predominantly (001) textured. Although several earlier works reported the occurrence of a phase transition between α and β phases by thermal treatment above 200°C [15,16], but such a phase transition was not observed in this work.

**Figure 3:**
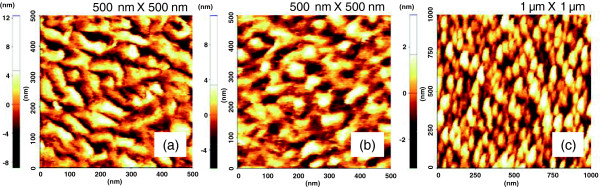
**AFM images of Si(001)/8 nm MgO/15 nm Fe/1.8 nm MgO/2 nm CuPc films.** (**a**) as grown, post-annealed at (**b**) 150°C and (**c**) 250°C.

Figure 3 shows the surface morphology of 20-nm-thick CuPc films (Figure 3a) before and after annealing at (Figure 3b) 150°C, and (Figure 3c) 250°C, respectively. Relatively smooth surfaces with root mean square (RMS) roughness of less than 2 nm were observed after the annealing up to 250°C. However, the RMS roughness became larger by annealing at the temperature higher than 250°C (Table 2). This is quite consistent with the strengthening of the [002] CuPc texture with increasing post-annealing temperature found by the XRD. This can simply reveal the enhancement of thermal stability and crystallinity of the CuPc layered structure due to the MgO(001) underlayer effect. From the AFM surface analysis, rather homogeneous roughness distributions for the vertical distance between the highest peak and the lowest valley were observed in the range from −4 to 4 nm.

**Table 2:** RMS roughness for CuPc films after post-annealing for 1 h under vacuum

**Post-annealing effect**		
**Heating *T *(°C)**	**RMS (nm)**	**Figure**
0 (as grown film)	2.05 ± 0.40	Figure 3a
150	1.79 ± 0.29	Figure 3b
250	0.89 ± 0.05	Figure 3c

**Figure 4:**
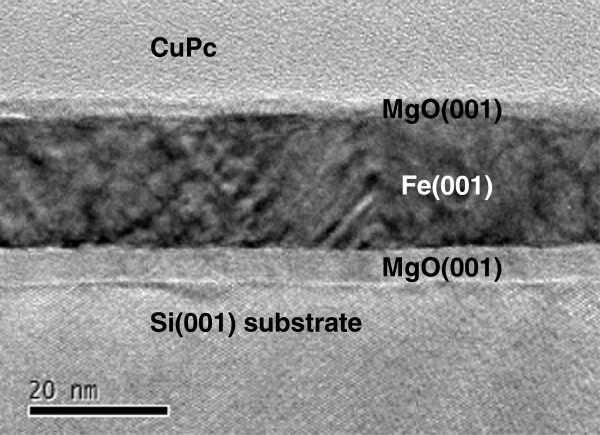
**Cross-sectional bright-field TEM image of Si**(**001**)/**8 nm MgO/15 nm Fe/1.8 nm MgO/20 nm CuPc film.**

Here, we report a hybrid system consisting of a highly qualified interface between the MgO/Fe/MgO(001) and the OSC CuPc. Figure 4 corresponds to the TEM image for the 20-nm-thick CuPc film grown at RT. The image shows, from bottom to top, the Si substrate, the 8-nm-thick MgO buffer layer, and the 10-nm-thick metallic Fe epilayer covered with the 1.8-nm-thick MgO. Note that the MgO/Fe/MgO(001) multilayers are well-crystallized, but some layer roughness is observable.

**Figure 5:**
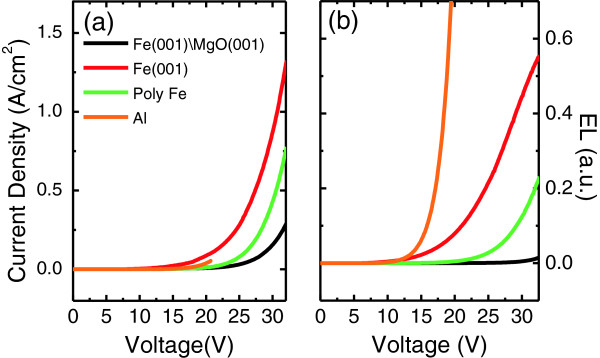
**Current density-voltage and luminance-voltage characteristics for TOLED devices with different anodes.** Al (blue, S1), Fe(100)/MgO(100) (green, S2), Fe(100) (red, S3), and poly-Fe (black, S4) anodes. More detailed information about each sample structure is given in Table 1.

Figure 5 shows the current density-voltage (*J*-*V*) (Figure 5a) and the luminance-voltage (*L*-*V*) (Figure 5b) characteristics for the TOLED devices with 10-nm-thick CuPc film prepared beyond the different anodes, such as Al (blue, S1), polycrystalline Fe (poly-Fe) (black, S4), Fe(001) with (green, S2) and without MgO(001) (red, S3). For more details of the TOLED structure, see Table 1.

**Figure 6:**
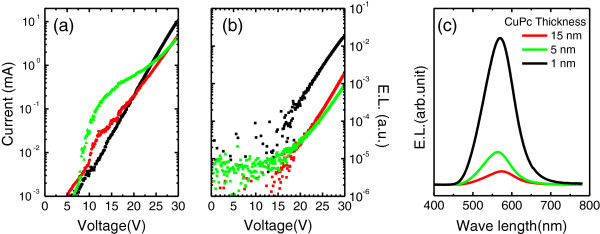
Current-voltage and luminance-voltage characteristics for TOLED devices.

When compared to the TOLED devices based on Al and poly-Fe and Fe(001) bottom electrodes, the threshold voltage for the TOLED based on the Fe/MgO(001) electrode increases significantly. More drastic increase in the driving voltage is also shown for that Fe/MgO(001)-based TOLED. The large driving voltage could be attributed to the larger work function of the MgO(001) layer (4.94 eV) [17] than that of Al (4.1 eV) [18] or poly-Fe (4.5 eV) [18]. Additionally, the effect of surface potential at the MgO(001)/CuPc interface could not be negligible. Since it was reported that charge injection barriers at metal/organic or oxide/organic interfaces affect the charge injection and recombination significantly [19-21], we also investigated the *IV* (Figure 6a) and *LV* (Figure 6b) characteristics for the Fe/MgO(001)-based TOLED with different CuPc thicknesses: 15 nm (red, S5), 5 nm (green, S6) and 1 nm (black, S7) as shown in Figure 6. The structure information of S5, 6 and 7 TOLED are given in Table 1. EL spectra of TOLED with different thickness of CuPc are shown in Figure 3c: red for S5, green for S6, and black for S7. When the thickness decreases from 15 to 1 nm, no remarkable change appeared in the threshold and driving voltages, but current and EL intensity increased obviously. A largely enhanced EL was observed in the TOLED with 1-nm-thick CuPc layer. To improve the TOLED device performance, further studies to optimize the device structure and fabrication conditions are required; the EL efficiency is far from perfect. However, our results suggest a new possibility to integrate spintronics with organic electronics: The use of the epitaxial thin MgO(001) layer is proposed not only to improve the performance and the stability of OLED, but also to inject the fully polarized spin current from the Fe/MgO(001) interface to the OSC layers [9]. Indeed, the enhanced thermal stability of a few-nanometer-thick CuPc films could result from the MgO(001) underlayer effect: The (002)-textured β phase of CuPc layer persists even after the vacuum annealing at 350°C. Significant work function alteration by inserting MgO(001) between ferromagnetic metal and the CuPc OSC layer could provide a wide versatility of device functionality. For example, polarized light could be generated by fully polarized spin injection through the Fe/MgO(001) interface. Thus, for future work, it is worth to study the magnetic field effect in this OLED device.

## Conclusions

In order to control the operation of an OLEDs, we investigated a new possibility of the use of thin MgO(001) layer between the ferromagnetic metal Fe(001)-OSC CuPc interface as a organic surface modifier. Remarkably enhanced thermal stability of CuPc films with smooth surface morphology (RMS roughness ≤ approximately 2 nm) was obtained up to the temperature of 350°C. In this work, the use of appropriate oxide layers could represent a new interface engineering technique for improving reliability and functionality in OSC devices. Based on the reliable Si(001)/MgO(001)/Fe(001)/MgO(001)/CuPc hybrid stack, the new TOLED structure was investigated for future organic spintronic device applications.

## Competing interests

The authors declare that they have no competing interests.

## Authors’ contributions

YJB and THK carried out the overall experiment and data analysis. NJL did the fabrication of OLEDs and the IVL experiments. HC and CL gave advice and helped for fabrication of OLEDs. LF and AH performed the TEM experiments. All authors read and approved the final manuscript.
